# Synthesis, Antifungal Activity and QSAR of Some Novel Carboxylic Acid Amides

**DOI:** 10.3390/molecules20034071

**Published:** 2015-03-04

**Authors:** Shijie Du, Huizhe Lu, Dongyan Yang, Hong Li, Xilin Gu, Chuan Wan, Changqing Jia, Mian Wang, Xiuyun Li, Zhaohai Qin

**Affiliations:** Department of Applied Chemistry, College of Science, China Agricultural University, Beijing 100193, China; E-Mails: dsj5216@163.com (S.D.); yangdy@cau.edu.cn (D.Y.); lihong129106@163.com (H.L.); david9351@126.com (X.G.); wanchuan2011@163.com (C.W.); cauchangqing@gmail.com (C.J.); woookooo@cau.edu.cn (M.W.); lixiuyun0115@163.com (X.L.)

**Keywords:** amide fungicides, boscalid, QSAR, SDHIs, molecular docking

## Abstract

A series of novel aromatic carboxylic acid amides were synthesized and tested for their activities against six phytopathogenic fungi by an *in vitro* mycelia growth inhibition assay. Most of them displayed moderate to good activity. Among them *N*-(2-(1*H*-indazol-1-yl)phenyl)-2-(trifluoromethyl)benzamide (**3c**) exhibited the highest antifungal activity against *Pythium aphanidermatum* (EC_50_ = 16.75 µg/mL) and *Rhizoctonia solani* (EC_50_ = 19.19 µg/mL), compared to the reference compound boscalid with EC_50_ values of 10.68 and 14.47 µg/mL, respectively. Comparative molecular field analysis (CoMFA) and comparative molecular similarity indices analysis (CoMSIA) were employed to develop a three-dimensional quantitative structure-activity relationship model for the activity of the compounds. In the molecular docking, a fluorine atom and the carbonyl oxygen atom of **3c** formed hydrogen bonds toward the hydroxyl hydrogens of TYR58 and TRP173.

## 1. Introduction

Amide fungicides, an old type of fungicide, still play an important role in the agricultural chemistry field nowadays [1]. Some excellent commercial fungicides including boscalid (BASF, 2003), tiadinil (Nihon Nohyaku Co., Ltd., 2004), fluxapyroxad (BASF, 2012) and benzovindiflupyr (Syngenta, 2012) belong to this class. Recently, many aspects of structural transformation and structure-activity relationship studies have been reported. Wen [2] and coworkers changed the biphenyl in the structure of boscalid to a diphenyl ether, and thus obtained target compounds with some extent of antifungal activities. Liu *et al.* synthesized a series of N-substituted pyridine amide fungicides exhibiting good activity to *Magnaporthe* *oryzae* and *Blumeria graminis* f. sp. *Tritici* [3]. Ye *et al.* changed the chlorine atom of boscalid to a methylthio or aromatic sulfur, and obtained some compounds with good activity to *Rhizoctonia solani* and *Sclerotinia sclerotiorum* [4].

**Figure 1:**
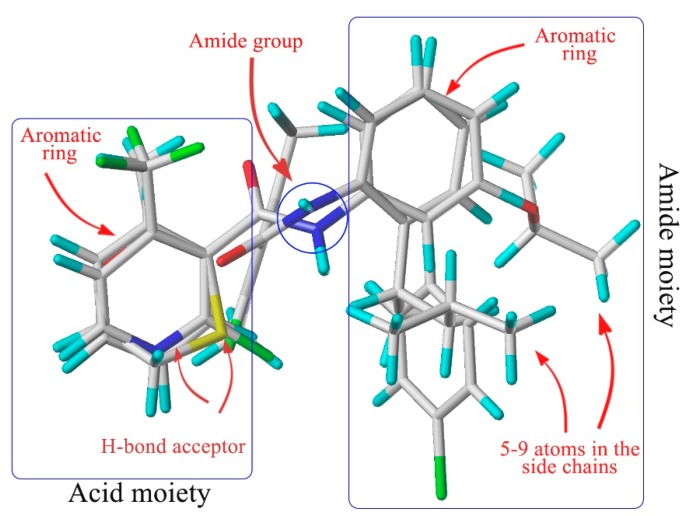
Structural alignment of the four kinds of SDHI fungicides (adapted from [8]).

Amide fungicides target the complex II (succinate dehydrogenase, SDH) in the mitochondrial respiratory chain [5]. As SDH inhibitors (SDHIs), they can inhibit the growth and even cause the death of pathogens by disrupting the mitochondrial tricarboxylic acid cycle [6] and then interfering with their respiration [7]. After analyzing the structure of the known SDHIs encompassing furametpyr, boscalid, fluxapyroxad, bixafen, penthiopyrad, penflufen, sedaxane, isopyrazam, benzovindiflupyr, we found that the aromatic amines share an auxiliary group with a five to nine atoms bridge and *ortho*-substituents. The alignment of the three-dimensional structures of carboxin, flutolanil, boscalid and penflufen (Figure 1) demonstrates that the common chemical features discussed above superimpose well, suggesting an identical binding mode at SDH. By summarizing the chemical structure of all commercial aromatic amides fungicides, Dehne found that they share common chemical features, which are essential for fungicidal activity, and they hence bind to their target in the same manner [8].

**Figure 2:**
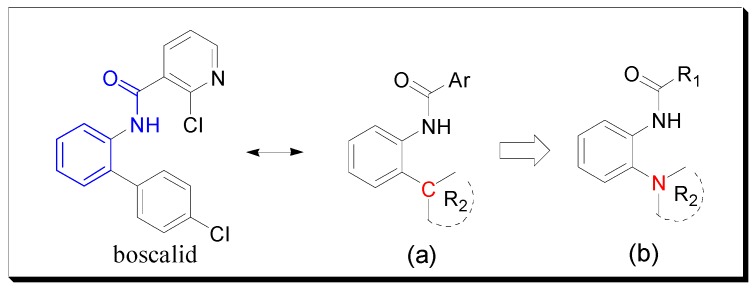
Design strategy of the target compounds. (**a**) Most general molecular structure. (**b**) The designed compound.

In most previous studies, the ortho of the amide group on the benzene ring is attached to a C atom (Figure 2a), which is an important part in some commercial fungicides. Based on bioisosterism [9,10] and computational docking experiments [8], we introduced a N atom to replace the C atom and thus designed and synthesized the target compounds (Figure 2b). Bioassays showed that some target molecules exhibited good antifungal activity and might be useful as potential lead compounds. 

## 2. Results and Discussion

### 2.1. Synthesis of Compounds

**Scheme 1:**
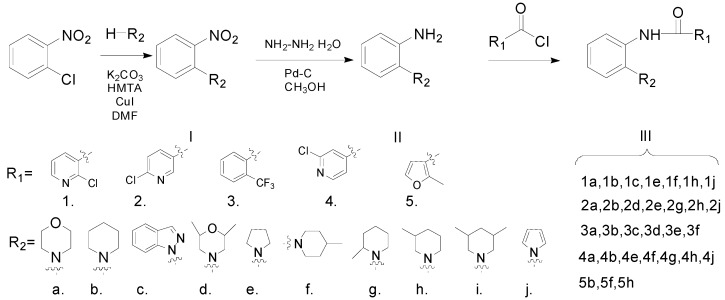
Synthetic pathway for the target compounds.

The synthetic route to the target compounds is shown in Scheme 1. The chlorine atom in 1-chloro-2-nitrobenzene was replaced by an amino group via an aromatic nucleophilic substitution reaction, giving the compound I. Then the nitro group in compound I was reduced with hydrazine hydrate to provide the key intermediates II, which were subsequently acylated to produce the target amides III.

### 2.2. In Vitro Antifungal Activity 

Table 1 summarizes the bioassay results. Overall, the target molecules showed different levels of antifungal activity. The inhibitory activity of the target molecules to *P. aphanidermatum* and *R. solani* were higher than against the other four fungi. Compounds **1c**, **1i**, **3c** exhibited wide spectrum antifungal activity and compound **1c** displayed excellent activities against *Colletotrichum orbiculare*, *Rhizoctonia solani*, *Pythium aphanidermatum* and *Botrytis cinerea*, with 60.43%, 75.19%, 74.14% and 79.27% inhibition rate, respectively. Moreover, some compounds showed highly specific pathogen activity, whereby compounds **1h** and **3e** exhibited activities against *Rhizoctonia solani* with 67.75% and 69.08% inhibition rates, respectively.

**Table 1:** *In vitro* antifungal activity of the target compounds against different phyto-pathogenic fungi (at 50 μg·mL^−1^).

Compd.	Inhibition rate ^a^ (%)					
	W. A. ^b^	B. B.	R. S.	P. I.	P. A.	B. C.
**1a**	11.11	30.53	17.56	33.15	21.17	12.50
**1b**	39.84	48.09	59.92	43.67	37.62	26.22
**1c**	60.43	27.10	75.19	18.89	74.14	79.27
**1e**	24.66	61.83	55.73	35.83	51.89	43.29
**1f**	56.10	55.98	47.71	42.50	60.58	31.30
**1h**	44.99	57.25	67.75	41.33	48.48	44.51
**1i**	51.22	54.71	74.24	45.07	42.89	51.83
**2a**	8.94	6.11	43.51	17.72	32.22	12.23
**2b**	46.61	25.70	51.53	41.33	15.27	14.23
**2d**	19.24	16.79	21.95	30.11	24.58	14.23
**2e**	23.31	11.20	39.69	27.07	49.41	*
**2j**	15.99	*	50.95	30.81	46.65	12.50
**2g**	50.95	40.71	58.78	34.32	42.27	21.34
**2h**	42.55	19.59	55.34	38.99	43.39	14.02
**3a**	32.93	31.30	42.56	31.04	42.89	11.59
**3b**	48.78	38.93	49.24	38.29	39.79	46.34
**3c**	47.97	56.49	75.57	36.42	84.17	49.59
**3d**	35.23	24.94	39.89	34.32	34.20	36.38
**3e**	43.63	28.24	69.08	33.61	38.18	23.78
**3f**	57.32	23.66	43.51	36.65	49.10	37.20
**4a**	11.38	18.32	36.45	17.72	10.61	*
**4b**	37.13	32.57	50.57	40.63	44.13	18.90
**4e**	27.10	32.44	50.95	30.11	57.79	*
**4j**	50.38	28.04	33.88	24.05	18.49	0.00
**4f**	*	37.09	21.04	31.30	39.73	63.33
**4g**	13.74	44.75	31.15	32.32	39.04	43.75
**4h**	*	47.54	24.59	29.52	38.58	20.00
**5b**	41.60	54.97	43.99	40.20	40.87	40.94
**5f**	35.11	52.65	60.11	32.32	40.87	49.58
**5h**	37.40	47.42	55.46	31.30	42.47	48.75
**Boscalid**	71.54	58.58	78.73	56.50	81.10	88.60

^a^ The tests were repeated three times, and the average inhibition rate is given. ^b^ W.A. = *Colletotrichum orbiculare*; B. B. = *Botryosphaeria berengeriana*; R. S. = *Rhizoctonia solani*; P. I. *= Phytophthora infestans* (Mont.) De Bary; P. A. = *Pythium aphanidermatum*; B. C*.* = *Botrytis cinerea*; * No antifungal activity

In order to study the structure-activity relationship of the target compounds further, we choose *Rhizoctonia solani* and *Pythium aphanidermatum* for precise virulence measurements. As we can see from Table 2, all of the synthetic compounds exhibited considerable antifungal effects toward the two fungi. The compounds **1c** and **3c** exhibited the best fungicidal activity against the two fungi, with corresponding EC_50_ values of 19.95 μg·mL^−1^, 17.99 μg·mL^−1^ and 16.75 μg·mL^−1^, 19.19 μg·mL^−1^, respectively. The antifungal activity of both compounds was close to that of the control compound boscalid. They also had broad antifungal spectrum, so they have potential value as secondary lead compounds for further research.

**Table 2:** Measured and predicted antifungal activities of target compounds against *Pythium aphanidermatum* and *Rhizoctonia solani.*

*Pythium aphanidermatum*				*Rhizoctonia* *solani*			
No.	EC_50_	pEC_50_	Predicted pEC_50_		EC_50_	pEC_50_	Predicted pEC_50_	
	(μg·mL^−1^)		CoMFA	CoMSIA	(μg·mL^−1^)		CoMFA	CoMSIA
**1a**	114.20	3.94	4.002	3.964	141.25	3.85	3.880	3.810
**1b**	74.90	4.13	3.971	3.967	74.99	4.12	4.037	4.023
**1c**	19.95	4.70	4.687	4.677	17.99	4.74	4.729	4.683
**1e**	82.78	4.08	4.170	4.042	77.98	4.11	4.080	4.065
**1f**	91.47	4.04	3.983	3.953	89.95	4.05	3.990	4.012
**1h**	94.23	4.03	4.019	3.993	78.16	4.11	4.035	4.032
**1i**	93.29	4.03	4.155	4.217	82.79	4.08	4.085	4.075
**2a**	87.12	4.06	3.973	3.972	108.52	3.96	3.856	3.820
**2b**	166.37	3.78	3.953	3.992	89.63	4.05	4.039	4.029
**2d**	117.20	3.93	4.013	3.953	163.22	3.79	3.830	3.839
**2e**	52.20	4.28	4.194	4.219	103.51	3.99	3.960	3.979
**2j**	56.23	4.25	4.240	4.240	50.12	4.30	4.301	4.323
**2g**	71.40	4.15	4.019	4.030	76.91	4.11	4.083	4.039
**2h**	79.90	4.10	3.997	4.016	84.92	4.07	4.050	4.037
**3a**	82.29	4.08	4.093	4.061	110.41	3.96	3.923	3.897
**3b**	80.49	4.09	4.121	4.174	87.9	4.06	3.990	4.002
**3c**	16.75	4.78	4.871	4.804	19.19	4.72	4.170	4.704
**3d**	77.04	4.11	4.051	4.033	90.57	4.04	4.006	4.051
**3e**	95.50	4.02	4.048	4.059	101.39	3.99	3.998	4.016
**3f**	69.35	4.16	4.111	4.140	102.09	3.99	3.954	3.991
**4a**	169.82	3.77	3.895	3.834	121.37	3.92	3.927	3.861
**4b**	84.10	4.08	3.895	3.931	85.9	4.07	4.001	4.026
**4e**	35.31	4.45	4.364	4.467	78.51	4.11	4.047	4.028
**4j**	79.43	4.10	4.231	4.227	53.7	4.27	4.316	4.350
**4f**	154.88	3.81	3.892	3.937	170.56	3.77	3.939	3.992
**4g**	141.25	3.85	3.971	3.965	101.39	3.99	4.018	4.034
**4h**	151.35	3.82	3.869	3.910	79.62	4.10	4.012	4.013
**5b**	31.29	4.50	4.388	4.433	103.04	3.99	3.957	4.054
**5f**	66.10	4.18	4.201	4.098	71.94	4.14	4.142	4.194
**5h**	41.68	4.38	4.315	4.405	95.72	4.02	4.022	3.969
**Boscalid**	10.68	4.97	4.960	4.938	14.47	4.84	4.896	4.860

From the high bioactivity of compounds **1c** and **3c** which contain an indazolyl group, we speculated that this group might have an essential influence on their biological activity. In recent years, indazole compounds have received much attention in the field of bioactive chemicals [11], especially in medicine, where they are known for anti-aggregatory and vasorelaxant activity, anticancer effects, and antimicrobial and antiparasitic properties [12]. Our studies now show that the indazolyl moiety also has potential use in agrochemicals.

### 2.3. Quantitative Structure-Activity Relationship (QSAR) Analyses

**Figure 3:**
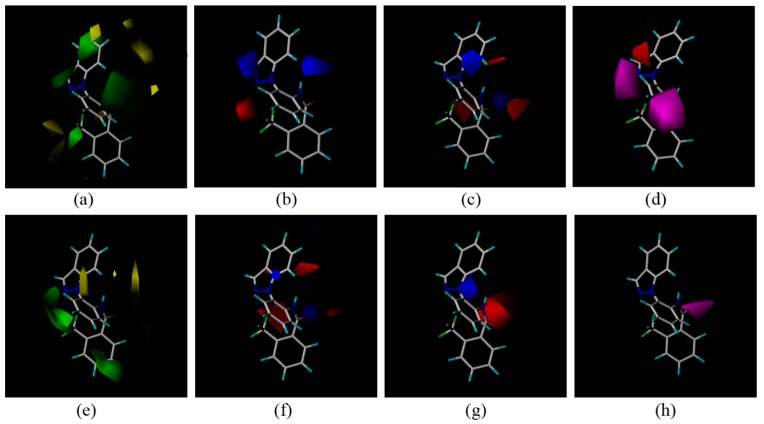
(**a**–**d**): Fitting of **3c** to *P. aphanidermatum.* (a) CoMFA steric field. (b) CoMFA electrostatic field. (c) CoMSIA electrostatic field. (d) CoMSIA H acceptor field. (**e**–**h**): fitting of **3c** to *R. solani.* (e) CoMFA steric field. (f) CoMFA electrostatic field. (g) CoMSIA electrostatic field. (h) CoMSIA H acceptor field. In the electrostatic field, the positively charged favored regions are shown in blue, and negatively charge favored regions are shown in red. In the sterically favored and disfavored region are shown in green and yellow respectively. In the hydrogen bond acceptor field, the favored regions are shown in magenta, and negatively charge favored regions are shown in red.

During biological screening, CoMFA and CoMSIA models of the new compounds were constructed using Sybyl 7.3 to find some clues about the structure-activity relationships. Compound **3c** was the template molecule used for constructing models for *P. aphanidermatum* activity. Some important parameters of the models were as follows: (A) the CoMFA model: q^2^ = 0.543 at 3 components, r^2^ = 0.814, F = 73.392; (B) the CoMSIA model: q^2^ = 0.502 at 3 components, r^2^ = 0.893, F = 75.480. For *R. solani*, some parameters of the models are as follows: (A) the CoMFA model: q^2^ = 0.61 at 3 components, r^2^ = 0.936, F = 73.446; (B) the CoMSIA model: q^2^ = 0.63 at 3 components, r^2^ = 0.888, F = 93.894. The three-dimensional contour maps of the CoMFA and CoMSIA are shown in Figure 3. 

In the three-dimensional contour maps of the CoMFA of compounds **3c** to *P. aphanidermatum* and *R. solani.* Some areas were covered with yellow blocks, which indicates that a large substituent in this position will likely reduce the biological activity of the compound. Green regions substantially covered the indazole and the benzene (Figure 3a,e), meaning that the introduction of greater steric hindrance groups at these positions was beneficial to improving the activity. In the CoMSIA and CoMFA electrostatic field, carbonyl and trifluoromethyl groups on the benzene ring were red blocks (Figure 3b,c,f,g), indicating that the introduction of negatively charged groups to the active position was beneficial for the antifungal activity. In the hydrogen acceptor field (Figure 3d,h), the results showed that more hydrogen bonds should improve the biological activity. As a hydrogen acceptor, the fluorine in the trifluoromethyl and the nitrogen atom in the pyrazole are helpful for forming hydrogen bonds, so taking together all this information, it can explain why **3c** showed better antifungal activity than the other compounds.

### 2.4. Molecular Docking

**Figure 4:**
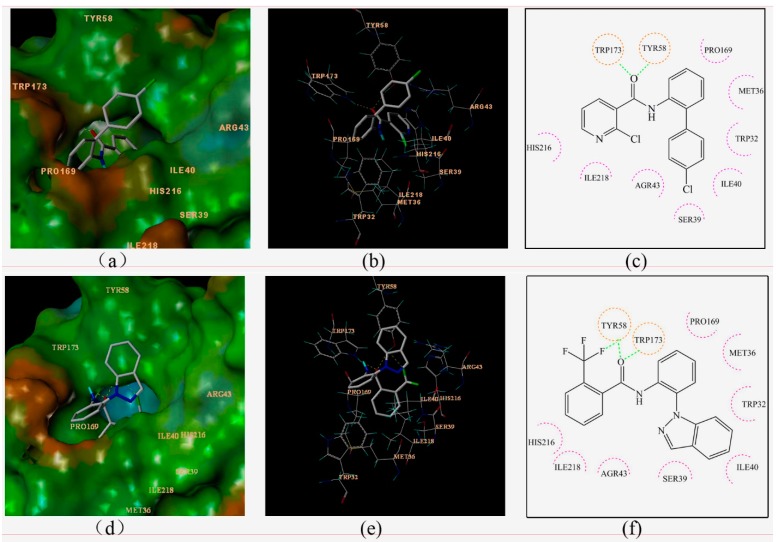
Surflex-Docking of boscalid to complex II. (**a**) Connolly surface of complex II with boscalid shown as a stick model. (**b**) Interaction of boscalid and amino acid residues near the ligands (3D diagram). (**c**) Interaction of boscalid and amino acid residues near the ligands (2D diagram). (**d**) Connolly surface of complex II with compounds **3c** shown as a stick model. (**e**) Interaction of compounds **3c** and amino acid residues near the ligands (3D diagram). (**f**) Interaction of compounds **3c** and amino acid residues near the ligands (2D diagram). The orange dotted line circles show the amino acids that participated in hydrogen bonding. The magenta dotted semicircle show the amino acids that participated in the van der Waals interactions. The hydrogen bond interactions are shown as green dotted lines.

In an effort to elucidate the possible antifungal mechanism of these compounds, molecular docking of compound **3c** to the binding site of SDH (pdb code: 2FBW [13,14]) pdb was performed. Boscalid was docked as a reference at the same time. The three-dimensional schematic diagrams clearly explained the possible optimal combination between the ligands and receptor protein (Figure 4).

Boscalid and compound **3c** adopted similar conformations and locations in the active site. The docking total scores were 5.25 and 6.87, respectively. They were both well bound to the receptor protein with their amino hydrogen toward the carboxyl oxygen of TRP173, and the carboxyl oxygen forming hydrogen bonds toward the hydroxyl hydrogen of TYR58. At the same time, an additional hydrogen bond was formed between the fluorine atom of compound **3c** and the hydroxyl hydrogen of TYR58. Two-dimensional diagrams are displayed as Figure 4c,f in which all of the amino acid residues TRP32, MET36, SER39, ILE40, AGR43, PRO169, HIS216, ILE218 interacted with the ligand, including weak interactions such as van der Waals interactions and polar interactions. Thus, a stable complex between compound **3c** and SDH was formed based on these interactions. The docking results were consistent with previous studies comparing boscalid and other SDHIs (such as 3-nitropropionic acid, 2-thenoyltrifluoroacetone, carboxin) [15].

Resistant fungal genotype analysis verified that most of those key residues involved in forming the binding cavity were related to resistance formation [16].

## 3. Experimental Section 

### 3.1. General Information

For all reactions, the solvents and chemical reagents were of analytical or synthetic grade obtained from Sinopharm Chemical Reagent Beijing Co., Ltd. (Beijing, China), and were used without purification. Column chromatography purification was performed using silica gel. Melting points were determined using a B-III microscope (Beijing Technical Instrument Co., Beijing China), and the thermometer was uncorrected. NMR spectra were obtained using an Avance DPX300 spectrometer (Bruker, Billerica, MA, USA) with tetramethylsilane (TMS) as the internal standard. High resolution mass spectrometry data were obtained with an Accurate-Mass-Q-TOF MS 6520 system equipped with an electrospray ionization (ESI) source (Agilent, Santa Clara, CA, USA).

### 3.2. Synthesis of Compounds

#### 3.2.1. Synthesis of Compounds I: 1-(2-Nitrophenyl) Piperidine 

In a 100 mL flask, 1-chloro-2-nitrobenzene (3.94 g, 0.025 mol), piperidine (2.55 g, 0.030 mol—we take R_1_ = piperidine as the example, the same method was used to synthesize and purify the rest of the reactants), potassium carbonate (6.9 g, 0.05 mol), HMTA (0.15 g), and CuI (0.2 g) were dissolved in DMF (50 mL), and the mixture was heated to 130 °C and refluxed for 24 h [17]. A same volume of water was then slowly added to the solution which was extracted with ethyl acetate (100 mL × 3). The combined organic layer was dried over anhydrous magnesium sulfate and evaporated under reduced pressure to give a crude product that was purified by column chromatography (EtOAc-PE = 10:1) to give the title compound as an orange powder, yield 75%; mp 76–77 °C; ^1^H-NMR (CDCl_3_) δ 7.74 (dd, *J* = 8.1, 1.6 Hz, 1H), 7.43 (ddd, *J* = 8.4, 7.3, 1.7 Hz, 1H), 7.11 (dd, *J* = 8.3, 1.2 Hz, 1H), 6.95 (ddd, *J* = 8.2, 7.3, 1.3 Hz, 1H), 3.09–2.95 (m, 4H), 1.84–1.66 (m, 4H), 1.64–1.52 (m, 2H).

#### 3.2.2. Synthesis of Compounds II: 2-(Piperidin-1-yl)aniline 

In an 100 mL three-necked flask equipped with a dropping funnel, 1-(2-nitrophenyl)piperidine (4.0 g) and ethanol (50 mL) were mixed and heated to reflux. Palladized charcoal (0.1 g, 5%, previously moistened with alcohol) was added. Next 80% hydrazine hydrate (15 mL) was added from a dropping funnel during 30 min. The reaction was continued for 8 h and then cooled [18,19]. The solid was filtered off and the filtrate was concentrated to give a crude product that was recrystallized from ethyl acetate and petroleum ether solution (15:1) to afford the title compound as a white powder*,* yield 95%; mp 48–50 °C; ^1^H-NMR (CDCl_3_) δ 7.02–6.95 (m, 1H), 6.95–6.86 (m, 1H), 6.82–6.63 (m, 2H), 3.97 (s, 2H), 2.83 (s, 4H), 1.77–1.63 (m, 4H), 1.57 (d, *J* = 5.0 Hz, 2H).

#### 3.2.3. Synthesis of Compounds III: 2-Chloro-*N*-(2-(piperidin-1-yl)phenyl)nicotinamide (**1b**) 

A solution of 2-(piperidin-1-yl)aniline (0.2 g, 0.025 mol), dichloromethane (25 mL) and triethylamine (0.5 mL) was added to a 50 mL flask and cooled to 0 °C in an ice bath. After ten minutes, 2-chloronicotinoyl chloride (0.22 g) was added [20,21]. The mixture was stirred for 30 min, and then concentrated under reduced pressure to give a crude product. The pure 2-chloro-*N*-(2-(piperidin-1-yl) phenyl) nicotinamide (**1b**) was obtained by column chromatography (EtOAc:PE = 8:1) purification. yield 88%; yellow powder; mp 76–77 °C; ^1^H-NMR (CDCl_3_) δ 9.77 (s, 1H, NH), 8.53 (dd, *J* = 4.8, 2.0 Hz, 2H, pyridyl-H), 8.22 (dd, *J* = 7.7, 2.0 Hz, 1H, pyridyl-H), 7.42 (dd, *J* = 7.7, 4.8 Hz, 1H, Ar-H), 7.25–7.06 (m, 3H, Ar-H), 2.93 (s, 2H, CH_2_), 2.71 (s, 2H, CH_2_), 1.88–1.64 (m, 2H, CH_2_), 1.44–1.22 (m, 2H, CH_2_), 0.97 (d, *J* = 6.4 Hz, 2H, CH_2_); ^13^C-NMR (CDCl_3_) δ 162.10, 150.81, 146.72, 142.92, 139.71, 133.20, 131.83, 125.24, 124.31, 122.62, 120.93, 119.21, 53.90, 26.44, 23.62; HRMS (ESI), *m/z* calcd for C_17_H_19_ClN_3_O (M+H)^+^ 316.1212, found 316.1211.

The following compounds were similarly prepared:

*2-Chloro-N-(2-morpholinophenyl)nicotinamide* (**1a**). Yield 78%; white powder; mp 81–83 °C; ^1^H-NMR (CDCl_3_) δ 9.81 (s, 1H, NH), 8.60 (dd, *J* = 8.0, 1.5 Hz, 1H, pyridyl-H), 8.54 (dd, *J* = 4.7, 2.0 Hz, 1H, pyridyl-H), 8.27 (dd, *J* = 7.7, 2.0 Hz, 1H, pyridyl-H), 7.44 (dd, *J* = 7.7, 4.7 Hz, 1H), 7.36–7.22 (m, 2H, Ar-H), 7.17 (td, *J* = 7.6, 1.6 Hz, 1H, Ar-H), 4.01–3.72 (m, 4H, CH_2_), 3.16–2.68 (m, 4H, CH_2_). ^13^C-NMR (CDCl_3_) δ 163.71, 150.50, 146.42, 142.74, 140.12, 133.31, 131.24, 125.14, 122.34, 122.43, 120.81, 119.62, 62.92, 55.43; HRMS (ESI), *m/z* calcd for C_16_H_16_ClN_3_O_2_ (M+H)^+^ 318.1004, found 318.1000.

*N-(2-(1H-Indazol-1-yl)phenyl)-2-chloronicotinamide* (**1c**). Yield 85%; yellow powder; mp 110–112 °C; ^1^H-NMR (CDCl_3_) δ 9.90 (s, 1H, NH), 8.63 (d, *J* = 8.2 Hz, 1H, pyridyl-H), 8.45 (dd, *J* = 4.8, 2.0 Hz, 1H, pyridyl-H), 8.24 (d, *J* = 0.9 Hz, 1H, pyridyl-H), 8.02 (dd, *J* = 7.6, 1.9 Hz, 1H, indazole-H), 7.81 (dt, *J* = 8.1, 1.0 Hz, 1H, Ar-H), 7.60 (dd, *J* = 3.0, 1.2 Hz, 1H, Ar-H), 7.57 (dd, *J* = 2.3, 1.3 Hz, 1H, Ar-H), 7.55–7.42 (m, 2H, Ar-H), 7.39–7.19 (m, 3H, Ar-H); ^13^C-NMR (CDCl_3_) δ 162.70, 150.85, 147.00, 139.76, 139.17, 135.98, 132.10, 131.40, 128.97, 128.26, 127.68, 124.82, 124.60, 124.16, 123.38, 122.34, 121.89, 121.14, 110.27; HRMS (ESI), *m/z* calcd for C_19_H_13_ClN_4_O (M+H)^+^ 349.0851, found 349.0851.

*2-Chloro-N-(2-(pyrrolidin-1-yl)phenyl)nicotinamide* (**1e**). Yield 86%; white powder; mp 136–138 °C; ^1^H-NMR (CDCl_3_) δ 9.66 (s, 1H, NH), 8.52 (dd, *J* = 4.7, 2.0 Hz, 1H, pyridyl-H), 8.47 (dd, *J* = 7.8, 1.9 Hz, 1H, pyridyl-H), 8.30 (dd, *J* = 7.7, 2.0 Hz, 1H, pyridyl-H), 7.42 (dd, *J* = 7.7, 4.7 Hz, 1H, Ar-H), 7.23 (d, *J* = 2.2 Hz, 1H, Ar-H), 7.16 (td, *J* = 7.1, 2.0 Hz, 2H, Ar-H), 3.05 (t, *J* = 6.6 Hz, 4H, CH_2_), 2.09–1.78 (m, 4H, CH_2_); ^13^C-NMR (CDCl_3_) δ 161.90, 150.82, 146.62, 140.45, 140.11, 133.13, 131.43, 124.63, 124.37, 122.68, 120.17, 120.10, 52.93, 24.20; HRMS (ESI), *m/z* calcd for C_16_H_16_ClN_3_O (M+H)^+^ 302.1052, found 302.1055.

*2-Chloro-N-(2-(4-methylpiperidin-1-yl)phenyl)nicotinamide* (**1f**). Yield 77%; white powder; mp 120–122 °C; ^1^H-NMR (CDCl_3_) δ 9.76 (s, 1H, NH), 8.56 (dd, *J* = 8.0, 1.6 Hz, 1H, pyridyl-H), 8.53 (dd, *J* = 4.8, 2.0 Hz, 1H, pyridyl-H), 8.21 (dd, *J* = 7.7, 2.0 Hz, 1H, pyridyl-H), 7.42 (dd, *J* = 7.7, 4.8 Hz, 1H, Ar-H), 7.18 (dddd, *J* = 15.1, 9.2, 6.7, 2.8 Hz, 3H, Ar-H), 2.91–2.73 (m, 4H, CH_2_), 1.76–1.64 (m, 4H, CH_2_), 1.45 (m, *J* = 6.5, 1H, CH), 0.86 (d, 2H, *J* = 6.5,CH3); ^13^C-NMR (CDCl_3_) δ 162.06, 150.83, 146.63, 142.57, 139.81, 133.29, 131.74, 125.20, 124.23, 122.61, 120.92, 119.19, 53.30, 34.73, 30.18, 21.56; HRMS (ESI), *m/z* calcd for C_18_H_20_ClN_3_O (M+H)^+^ 330.1363, found 330.1368.

*2-Chloro-N-(2-(3-methylpiperidin-1-yl)phenyl)nicotinamide* (**1h**). Yield 76%; white powder; mp 92–94 °C; ^1^H-NMR (CDCl_3_) δ 9.71 (s, 1H, NH), 8.56 (dd, *J* = 7.9, 1.4 Hz, 1H, pyridyl-H), 8.52 (dd, *J* = 4.8, 2.0 Hz, 1H, pyridyl-H), 8.20 (dd, *J* = 7.7, 2.0 Hz, 1H, pyridyl-H), 7.41 (dd, *J* = 7.7, 4.8 Hz, 1H, Ar-H), 7.26–7.08 (m, 3H, Ar-H), 3.01–2.82 (m, 2H, CH_2_), 2.63 (td, *J* = 11.4, 2.9 Hz, 1H, CH), 2.42–2.22 (m, 1H, CH), 1.89–1.61 (m, 4H, CH_2_), 1.03 (dd, *J* = 11.8, 2.5 Hz, 1H, CH), 0.88 (d, *J* = 6.5 Hz, 3H, CH_3_); ^13^C-NMR (CDCl_3_) δ 162.13, 150.79, 146.62, 142.54, 139.58, 133.24, 131.86, 125.18, 124.24, 122.60, 120.93, 119.13, 61.05, 53.29, 32.19, 31.64, 25.85, 19.06; HRMS (ESI), *m/z* calcd for C_18_H_20_ClN_3_O (M+H)^+^ 330.1362, found 330.1368.

*2-Chloro-N-(2-(3-methylpiperidin-1-yl)phenyl)nicotinamide* (**1i**). Yield 83%; white powder; mp 90–92 °C; ^1^H-NMR (CDCl_3_) δ 9.71 (s, 1H, NH), 8.61–8.54 (m, 1H, pyridyl-H), 8.52 (dd, *J* = 4.8, 2.0 Hz, 1H, pyridyl-H), 8.20 (dd, *J* = 7.7, 2.0 Hz, 1H, pyridyl-H), 7.42 (dd, *J* = 7.7, 4.8 Hz, 1H, Ar-H), 7.25–7.08 (m, 3H, Ar-H), 2.90 (d, *J* = 7.2 Hz, 2H, CH_2_), 2.24 (t, *J* = 11.0 Hz, 2H, CH), 1.89–1.72 (m, 4H, CH_2_), 0.88 (d, *J* = 6.4 Hz, 6H, CH_3_); ^13^C-NMR (CDCl_3_) δ 162.12, 150.79, 146.56, 142.28, 139.62, 133.25, 131.87, 125.20, 124.23, 122.61, 120.93, 120.82, 119.14, 60.62, 41.47, 31.75, 19.04; HRMS (ESI), *m/z* calcd for C_19_H_22_ClN_3_O (M+H)^+^ 344.1527, found 344.1524.

*6-Chloro-N-(2-morpholinophenyl)nicotinamide* (**2a**). Yield 75%; white powder; mp 133–135 °C; ^1^H-NMR (CDCl_3_) δ 9.60 (s, 1H, NH), 9.09 (ddd, *J* = 9.5, 2.4, 0.7 Hz, 1H, pyridyl-H), 8.92 (dd, *J* = 2.5, 0.7 Hz, 1H, pyridyl-H), 8.68–8.44 (m, 1H, pyridyl-H), 8.39–8.17 (m, 2H, Ar-H), 7.54 (ddd, *J* = 8.3, 6.4, 0.7 Hz, 1H, Ar-H), 7.22–7.11 (m, 1H, Ar-H), 4.07–3.58 (m, 4H, CH_2_), 3.10–2.69 (m, 4H, CH_2_). ^13^C-NMR (CDCl_3_) δ162.51, 154.26, 147.47, 140.62, 137.81, 132.48, 129.62, 124.61, 124.43, 124.30, 120.21, 119.03, 66.91, 53.24; HRMS (ESI), *m/z* calcd for C_16_H_16_ClN_3_O_2_ (M+H)^+^ 318.1004, found 318.1000.

*6-Chloro-N-(2-(piperidin-1-yl)phenyl)nicotinamide* (**2b**). Yield 76%; white powder; mp 126–128 °C; ^1^H-NMR (CDCl_3_) δ 9.70 (s, 1H, NH), 8.92 (dd, *J* = 2.5, 0.6 Hz, 1H, pyridyl-H), 8.50 (dd, *J* = 7.9, 1.5 Hz, 1H, pyridyl-H), 8.28 (dd, *J* = 8.3, 2.5 Hz, 1H, pyridyl-H), 7.51 (dd, *J* = 8.3, 0.7 Hz, 1H, Ar-H), 7.24–7.05 (m, 3H, Ar-H), 2.92–2.71 (m, 4H, CH_2_), 1.84–1.69 (m, 4H, CH_2_), 1.61 (d, *J* = 16.7 Hz, 2H, CH_2_). ^13^C-NMR (CDCl_3_) δ 162.17, 151.01, 147.62, 142.52, 141.21, 134.12, 132.20, 126.24, 124.81, 122.42, 121.05, 120.11, 55.87, 27.64, 24.66; HRMS (ESI), *m/z* calcd for C_17_H_18_ClN_3_O (M+H)^+^ 316.1211, found 316.1205.

*6-Chloro-N-(2-(2,6-dimethylmorpholino)phenyl)nicotinamide* (**2d**). Yield 77%; yellow powder; mp 147–148 °C; ^1^H-NMR (CDCl_3_) δ 9.65 (s, 1H, NH), 8.88 (d, *J* = 2.5 Hz, 1H, pyridyl-H), 8.58–8.46 (m, 1H, pyridyl-H), 8.25 (dd, *J* = 8.3, 2.5 Hz, 1H, pyridyl-H), 7.51 (d, *J* = 8.3 Hz, 1H, Ar-H), 7.17 (ddd, *J* = 15.3, 9.6, 4.6 Hz, 3H, Ar-H), 3.84 (dtd, *J* = 12.6, 6.3, 4.2 Hz, 2H, CH_2_), 2.82 (d, *J* = 10.8 Hz, 2H, CH_2_), 2.69–2.45 (m, 2H, CH), 1.23 (d, *J* = 6.3 Hz, 6H, CH_3_). ^13^C-NMR (CDCl_3_) δ162.45, 152.37, 146.31, 140.60, 137.75, 133.52, 131.61, 125.18, 124.43, 124.14, 121.41, 120.13, 69.14, 56.57, 17.56; HRMS (ESI), *m/z* calcd for C_18_H_20_ClN_3_O_2_ (M+H)^+^ 346.1317, found 346.1312.

*6-Chloro-N-(2-(pyrrolidin-1-yl)phenyl)nicotinamide* (**2e**). Yield 81%; white powder; mp 102–104 °C; ^1^H-NMR (CDCl_3_) δ 9.28 (s, 1H, NH), 8.85 (d, *J* = 2.1 Hz, 1H, pyridyl-H), 8.36 (d, *J* = 6.9 Hz, 1H, pyridyl-H), 8.22 (dd, *J* = 8.3, 2.4 Hz, 1H, pyridyl-H), 7.48 (d, *J* = 8.3 Hz, 1H, Ar-H), 7.33–7.01 (m, 3H, Ar-H), 3.05 (d, *J* = 5.7 Hz, 4H, CH_2_), 2.20–1.84 (m, 4H, CH_2_); ^13^C-NMR (CDCl_3_) δ 161.56, 154.06, 147.43, 140.32, 137.81, 132.39, 129.60, 124.60, 124.33, 124.20, 120.11, 119.63, 52.53, 24.24; HRMS (ESI), *m/z* calcd for C_16_H_16_ClN_3_O (M+H)^+^ 302.1051, found 302.1055.

*6-Chloro-N-(2-(2-methylpiperidin-1-yl)phenyl)nicotinamide* (**2g**) Yield 82%; white powder; mp 100–102 °C; ^1^H-NMR (CDCl_3_) δ 10.22 (s, 1H, NH), 8.92 (dd, *J* = 2.5, 0.7 Hz, 1H, pyridyl-H), 8.55 (dd, *J* = 8.0, 1.4 Hz, 1H, pyridyl-H), 8.28 (dd, *J* = 8.3, 2.5 Hz, 1H, pyridyl-H), 7.51 (dd, *J* = 8.3, 0.7 Hz, 1H, Ar-H), 7.26 (dq, *J* = 4.3, 1.6 Hz, 2H, Ar-H), 7.13 (td, *J* = 7.6, 1.6 Hz, 1H, Ar-H), 3.04–2.81 (m, 2H, CH_2_), 2.69 (td, *J* = 11.6, 2.6 Hz, 1H, CH), 1.98–1.74 (m, 2H, CH_2_), 1.71–1.32 (m, 4H, CH_2_), 0.81 (d, *J* = 6.2 Hz, 3H, CH_3_); ^13^C-NMR (CDCl_3_) δ 161.11, 154.05, 147.26, 140.40, 137.96, 134.96, 129.73, 125.94, 124.41, 123.86, 122.59, 118.36, 56.29, 55.30, 35.37, 27.23, 24.18, 19.97; HRMS (ESI), *m/z* calcd for C_18_H_20_ClN_3_O (M+H)^+^ 330.1363, found 330.1368.

*6-Chloro-N-(2-(3-methylpiperidin-1-yl)phenyl)nicotinamide* (**2h**) Yield 85%; white powder; mp 90–91 °C; ^1^H-NMR (CDCl_3_) δ 9.71 (s, 1H, NH), 8.90 (dd, *J* = 2.5, 0.6 Hz, 1H, pyridyl-H), 8.69–8.42 (m, 1H, pyridyl-H), 8.27 (dd, *J* = 8.3, 2.5 Hz, 1H, pyridyl-H), 7.51 (dd, *J* = 8.3, 0.7 Hz, 1H, Ar-H), 7.25–7.06 (m, 3H, Ar-H), 3.01–2.87 (m, 2H, CH_2_), 2.77–2.57 (m, 1H, CH_2_), 2.49–2.29 (m, 1H, CH_2_), 1.92–1.81 (m, 2H, CH_2_), 1.80–1.57 (m, 2H, CH_2_), 1.09 (d, *J* = 9.8 Hz, 1H, CH), 0.93 (d, *J* = 6.5 Hz, 3H, CH_3_); ^13^C-NMR (CDCl_3_) δ 161.31, 154.09, 147.25, 142.27, 137.96, 132.96, 129.68, 125.31, 124.41, 124.10, 120.70, 118.82, 60.75, 53.21, 32.33, 32.19, 26.40, 19.10; HRMS (ESI), *m/z* calcd for C_18_H_20_ClN_3_O (M+H)^+^ 330.1363, found 330.1368.

*N-(2-(1H-Pyrrol-1-yl)phenyl)-6-chloronicotinamide* (**2j**). Yield 81%; yellow powder; mp 150–152 °C; ^1^H-NMR (CDCl_3_) δ 8.60–8.50 (m, 2H, pyridyl-H), 7.95 (dd, *J* = 8.3, 2.6 Hz, 1H, pyridyl-H), 7.63 (s, 1H, NH), 7.53–7.35 (m, 3H, Ar-H), 7.31–7.20 (m, H, Ar-H), 6.84 (t, *J* = 2.1 Hz, 2H, pyrrole-H), 6.47 (t, *J* = 2.1 Hz, 2H, pyrrole-H). ^13^C-NMR (CDCl_3_) δ 161.22, 154.12, 147.40, 141.32, 138.12, 132.34, 129.42, 124.57, 124.23, 124.05, 120.19, 120.86, 119.41, 111.27; HRMS (ESI), *m/z* calcd for C_16_H_12_ClN_3_O (M+H)^+^ 298.0742, found 298.0742.

*N-(2-Morpholinophenyl)-2-(trifluoromethyl)benzamide* (**3a**). Yield 86%; yellow powder; mp 112–114 °C; ^1^H-NMR (300 MHz, CDCl_3_) δ 8.93 (s, 1H, NH), 8.54 (d, *J* = 8.1 Hz, 1H, Ar-H), 7.78 (d, *J* = 7.5 Hz, 1H, Ar-H), 7.65 (dd, *J* = 4.8, 3.1 Hz, 3H, Ar-H), 7.26–7.21 (m, 2H, Ar-H), 7.19–7.11 (m, 1H, Ar-H), 3.88–3.60 (m, 4H, CH_2_), 2.96–2.76 (m, 4H, CH_2_); ^13^C-NMR (CDCl_3_) δ 165.17, 140.87, 135.92, 133.36, 132.00, 129.87, 128.14, 126.61, 126.37, 126.31, 125.81, 124.26, 120.81, 119.54, 67.10, 52.47; HRMS (ESI), *m/z* calcd for C_18_H_17_F_3_N_2_O_2_ (M+H)^+^ 351.1310, found 351.1315.

*N-(2-(Piperidin-1-yl)phenyl)-2-(trifluoromethyl)benzamide* (**3b**). Yield 77%; brown powder; mp 80–82 °C; ^1^H-NMR (CDCl_3_) δ 8.92 (s, 1H, NH), 8.50 (dd, *J* = 6.8, 2.8 Hz, 1H, Ar-H), 7.77 (d, *J* = 7.7 Hz, 1H, Ar-H), 7.71–7.53 (m, 3H, Ar-H), 7.24–7.06 (m, 3H, Ar-H), 2.98–2.61 (m, 4H, CH_2_), 1.78–1.37 (m, 6H, CH_2_); ^13^C-NMR (CDCl_3_) δ 165.18, 142.68, 133.19, 131.91, 129.74, 127.89, 126.48, 126.41, 126.35, 124.91, 124.04, 120.45, 119.23, 53.56, 26.43, 23.60; HRMS (ESI), *m/z* calcd for C_19_H_19_F_3_N_2_O (M+H)^+^ 349.1518, found 349.1522.

*N-(2-(1H-Indazol-1-yl)phenyl)-2-(trifluoromethyl)benzamide* (**3c**). Yield 76%; yellow powder; mp 139–141 °C; ^1^H-NMR (CDCl_3_) δ 9.55 (s, 1H, NH), 8.68-8.50 (m, 1H, Ar-H), 8.15 (d, *J* = 0.8 Hz, 1H, lndazole-H), 7.83–7.76 (m, 1H, Ar-H), 7.74–7.67 (m, 1H, Ar-H), 7.63 (dd, *J* = 8.5, 0.9 Hz, 1H, Ar-H), 7.60–7.51 (m, 3H, Ar-H), 7.51–7.41 (m, 3H, Ar-H), 7.36–7.28 (m, 1H, Ar-H), 7.27–7.21 (m, 1H, Ar-H); ^13^C-NMR (CDCl_3_) δ 165.39, 139.66, 135.65, 132.12, 131.77, 129.89, 128.95, 128.15, 127.71, 127.69, 127.63, 126.46, 126.40, 124.66, 124.22, 124.11, 123.54, 121.90, 121.08, 110.47; HRMS (ESI), *m/z* calcd for C_21_H_14_F_3_N_3_O (M+H)^+^ 382.115, found 382.1162.

*N-(2-(2,6-Dimethylmorpholino)phenyl)-2-(trifluoromethyl)benzamide* (**3d**). Yield 86%; yellow powder; mp 100–102 °C; ^1^H-NMR (CDCl_3_) δ 8.86 (s, 1H, NH), 8.59–8.42 (m, 1H, Ar-H), 7.77 (d, *J* = 7.4 Hz, 1H, Ar-H), 7.72–7.57 (m, 3H, Ar-H), 7.25–7.03 (m, 3H, Ar-H), 3.73–3.54 (m, 2H, CH_2_), 2.77 (d, *J* = 10.8 Hz, 2H, CH_2_), 2.60–2.36 (m, 2H, CH_2_), 1.17 (d, *J* = 6.3 Hz, 6H, CH_3_); ^13^C-NMR (CDCl_3_) δ 165.18, 140.62, 133.29, 131.98, 129.84, 128.07, 127.00, 126.36, 126.30, 125.68, 124.27, 120.77, 119.60, 72.04, 57.97, 18.54; HRMS (ESI), *m/z* calcd for C_20_H_21_F_3_N_2_O_2_ (M+H)^+^ 379.1621, found 379.1628.

*N-(2-(Pyrrolidin-1-yl)phenyl)-2-(trifluoromethyl)benzamide* (**3e**). Yield 88%; white powder; mp 130–131 °C; ^1^H-NMR (MHz, CDCl_3_) δ 8.61 (s, 1H, NH), 8.41 (dd, *J* = 7.6, 1.8 Hz, 1H, Ar-H), 7.77 (d, *J* = 7.5 Hz, 1H, Ar-H), 7.63 (dd, *J* = 12.8, 4.1 Hz, 3H, Ar-H), 7.14 (ddd, *J* = 14.2, 6.8, 2.0 Hz, 3H, Ar-H), 3.01 (t, *J* = 6.6 Hz, 4H, CH_2_), 1.98–1.77 (m, 4H, CH_2_); ^13^C-NMR (CDCl_3_) δ 165.36, 140.42, 132.78, 131.90, 129.79, 129.69, 128.13, 126.36, 126.29, 124.55, 124.18, 120.58, 119.66, 52.59, 24.15; HRMS (ESI), *m/z* calcd for C_18_H_17_F_3_N_2_O (M+H)^+^ 335.1361, found 335.1366.

*N-(2-(4-Methylpiperidin-1-yl)phenyl)-2-(trifluoromethyl)benzamide* (**3f**). Yield 74%; white powder; mp 77–79 °C; ^1^H-NMR (CDCl_3_) δ 8.88 (s, 1H, NH), 8.63-8.39 (m, 1H, Ar-H), 7.78 (d, *J* = 7.5 Hz, 1H, Ar-H), 7.72–7.55 (m, 3H, Ar-H), 7.24–7.04 (m, 3H, Ar-H), 2.94 (d, *J* = 11.8 Hz, 2H, CH_2_), 2.69 (dd, *J* = 11.8, 2.3 Hz, 2H, CH_2_), 1.71 (d, *J* = 10.8 Hz, 2H, CH_2_), 1.54–1.41 (m, 1H, CH), 1.28–1.04 (m, 2H, CH_2_), 0.92 (d, *J* = 6.5 Hz, 3H, CH_3_); ^13^C-NMR (CDCl_3_) δ 165.19, 142.43, 133.18, 131.91, 129.75, 127.90, 126.89, 126.43, 126.36, 126.30, 124.91, 124.05, 120.43, 119.30, 52.94, 34.78, 30.13, 21.49; HRMS (ESI), *m/z* calcd for C_20_H_21_F_3_N_2_O (M+H)^+^ 363.1675, found 363.1675. 

*2-Chloro-N-(2-morpholinophenyl)isonicotinamide* (**4a**). Yield 76%; yellow powder; mp 134–136 °C; ^1^H-NMR (CDCl_3_) δ 9.62 (s, 1H, NH), 8.61 (dd, *J* = 5.1, 0.6 Hz, 1H, pyridyl-H), 8.51 (d, *J* = 8.0 Hz, 1H, pyridyl-H), 7.81 (d, *J* = 0.8 Hz, 1H, pyridyl-H), 7.71–7.60 (m, 1H, Ar-H), 7.32–7.00 (m, 3H, Ar-H), 4.07–3.78 (m, 4H, CH_2_), 3.13–2.45 (m, 4H, CH_2_). ^13^C-NMR (CDCl_3_) δ161.51, 152.23, 150.35, 146.14, 141.24, 132.27, 125.18, 124.34, 121.56, 120.54, 120.24, 119.07, 63.32, 56.40; HRMS (ESI), *m/z* calcd for C_16_H_16_ClN_3_O_2_ (M+H)^+^ 318.1004, found 318.1005.

*2-Chloro-N-(2-(piperidin-1-yl)phenyl)isonicotinamide* (**4b**). Yield 84%; white powder; mp 90–92 °C; ^1^H-NMR (CDCl_3_) δ 9.71 (s, 1H, NH), 8.60 (d, *J* = 5.1 Hz, 1H, pyridyl-H), 8.55–8.45 (m, 1H, pyridyl-H), 7.83 (d, *J* = 0.8 Hz, 1H, pyridyl-H), 7.66 (dd, *J* = 5.1, 1.5 Hz, 1H, Ar-H), 7.25–7.09 (m, 3H, Ar-H), 2.93–2.80 (m, 4H, CH_2_), 1.78 (dd, *J* = 10.7, 5.4 Hz, 4H, CH_2_), 1.63 (d, *J* = 18.5 Hz, 2H, CH_2_); ^13^C-NMR (CDCl_3_) δ 160.80, 152.53, 150.40, 145.08, 142.64, 132.57, 125.26, 124.43, 121.86, 120.67, 119.04, 118.89, 53.64, 26.94, 23.65; HRMS (ESI), *m/z* calcd for C_17_H_18_ClN_3_O (M+H)^+^ 316.1211, found 316.1211.

*2-Chloro-N-(2-(pyrrolidin-1-yl)phenyl)isonicotinamide* (**4e**). Yield 80%; yellow powder; mp 108–110 °C; ^1^H-NMR (CDCl_3_) δ 9.28 (s, 1H, NH), 8.58 (d, *J* = 5.1 Hz, 1H, pyridyl-H), 8.36 (d, *J* = 7.3 Hz, 1H, pyridyl-H), 7.77 (s, 1H, pyridyl-H), 7.62 (d, *J* = 5.1 Hz, 1H, Ar-H), 7.31–7.11 (m, 3H, Ar-H), 3.07 (t, *J* = 6.1 Hz, 4H, CH_2_), 2.20–1.84 (m, 4H, CH_2_). ^13^C-NMR (CDCl_3_) δ 160.45, 151.83, 150.35, 145.45, 142.26, 132.37, 125.26, 124.85, 121.57, 121.16, 119.04, 118.89, 53.35, 23.83; HRMS (ESI), *m/z* calcd for C_16_H_16_ClN_3_O (M+H)^+^ 302.1055, found 302.1054.

*2-Chloro-N-(2-(4-methylpiperidin-1-yl)phenyl)isonicotinamide* (**4f**). Yield 85%; white powder; mp 80–82 °C; ^1^H-NMR (CDCl_3_) δ 9.72 (s, 1H, NH), 8.60 (dd, *J* = 5.1, 0.7 Hz, 1H, pyridyl-H), 8.48 (dd, *J* = 7.9, 1.5 Hz, 1H, pyridyl-H), 7.82 (dd, *J* = 1.5, 0.7 Hz, 1H, pyridyl-H), 7.68 (dd, *J* = 5.1, 1.5 Hz, 1H, Ar-H), 7.26–7.09 (m, 3H, Ar-H), 2.97 (d, *J* = 12.0 Hz, 2H, CH_2_), 2.76 (td, *J* = 11.7, 2.3 Hz, 2H, CH_2_), 1.87 (d, *J* = 13.1 Hz, 2H, CH_2_), 1.71–1.53 (m, 1H, CH), 1.46-1.20 (m, 2H, CH_2_), 1.05 (d, *J* = 6.5 Hz, 3H, CH_3_). ^13^C-NMR (CDCl_3_) δ 160.41, 152.37, 150.25, 145.42, 140.46, 134.74, 125.72, 124.24, 122.59, 121.63, 119.44, 118.72, 56.52, 35.71, 33.20, 20.14; HRMS (ESI), *m/z* calcd for C_18_H_20_ClN_3_O (M+H)^+^ 330.1368, found 330.1368.

*2-Chloro-N-(2-(2-methylpiperidin-1-yl)phenyl)isonicotinamide* (**4g**). Yield 78%; yellow powder; mp 82–84 °C; ^1^H-NMR (CDCl_3_) δ 10.25 (s, 1H, NH), 8.60 (dd, *J* = 5.1, 0.7 Hz, 1H, pyridyl-H), 8.54 (dd, *J* = 7.9, 1.3 Hz, 1H, pyridyl-H), 7.83 (dd, *J* = 1.5, 0.7 Hz, 1H, pyridyl-H), 7.66 (dd, *J* = 5.1, 1.5 Hz, 1H, Ar-H), 7.31–7.20 (m, 2H, Ar-H), 7.14 (td, *J* = 7.6, 1.6 Hz, 1H, Ar-H), 3.03–2.82 (m, 2H, CH_2_), 2.71 (td, *J* = 11.5, 2.5 Hz, 1H, CH), 1.98–1.87 (m, 2H, CH_2_), 1.65–1.35 (m, 4H, CH_2_), 0.82 (d, *J* = 6.2 Hz, 3H, CH_3_); ^13^C-NMR (CDCl_3_) δ 160.56, 152.53, 150.39, 145.14, 140.50, 134.65, 125.96, 124.19, 122.66, 121.89, 119.00, 118.40,56.27, 55.22, 35.43, 27.27, 24.20, 19.96; HRMS (ESI), *m/z* calcd for C_18_H_20_ClN_3_O (M+H)^+^ 330.1367, found 330.1368.

*2-Chloro-N-(2-(3-methylpiperidin-1-yl)phenyl)isonicotinamide* (**4h**). Yield 83%; white powder; mp 81–83 °C; ^1^H-NMR (CDCl_3_) δ 9.70 (s, 1H, NH), 8.59 (d, *J* = 5.1 Hz, 1H, pyridyl-H), 8.49 (d, *J* = 8.2 Hz, 1H, pyridyl-H), 7.82 (s, 1H, pyridyl-H), 7.65 (dd, *J* = 5.1, 1.4 Hz, 1H, Ar-H), 7.24–7.05 (m, 3H, Ar-H), 2.93 (d, *J* = 11.4 Hz, 2H, CH_2_), 2.67 (td, *J* = 11.4, 2.2 Hz, 1H, CH_2_), 2.54–2.31 (m, 1H, CH_2_), 1.95–1.83 (m, 2H, CH_2_), 1.69 (d, *J* = 12.7 Hz, 2H, CH_2_), 1.20–1.04 (m, 1H, CH), 0.96 (d, *J* = 6.6 Hz, 3H, CH_3_); ^13^C-NMR (CDCl_3_) δ 160.86, 152.50, 150.39, 145.11, 142.35, 132.61, 125.30, 124.45, 121.84, 120.71, 119.05, 118.95, 60.66, 53.24, 32.28, 32.15, 26.32, 19.05; HRMS (ESI), *m/z* calcd for C_18_H_20_ClN_3_O (M+H)^+^ 330.1367, found 330.1368.

*N-(2-(1H-Pyrrol-1-yl)phenyl)-2-chloroisonicotinamide* (**4j**). Yield 82%; yellow powder; mp 120–122 °C; ^1^H-NMR (CDCl_3_) δ 8.51 (dd, *J* = 12.6, 6.2 Hz, 2H, pyridyl-H), 7.66 (s, 1H, NH), 7.53 (t, *J* = 2.7 Hz, 1H, pyridyl-H), 7.50–7.37 (m, 2H, Ar-H), 7.35–7.20 (m, 2H, Ar-H), 6.85 (t, *J* = 2.1 Hz, 2H, pyrrole-H), 6.50 (t, *J* = 2.1 Hz, 2H, pyrrole-H). ^13^C-NMR (CDCl_3_) δ 162.11, 152.43, 151.42, 146.55, 142.24, 132.14, 126.20, 124.53, 121.19, 120.16, 119.04, 118.89, 118.42, 110.63; HRMS (ESI), *m/z* calcd for C_16_H_12_ClN_3_O (M+H)^+^ 298.0742, found 298.0742.

*2-Methyl-N-(2-(piperidin-1-yl)phenyl)furan-3-carboxamide* (**5b**). Yield 80%; yellow paste; ^1^H-NMR (CDCl_3_) δ 9.12 (s, 1H, NH), 8.48 (d, *J* = 8.1 Hz, 1H, Ar-H), 7.33 (d, *J* = 2.1 Hz, 1H, furan-H), 7.22–7.11 (m, 2H, Ar-H), 7.06 (dd, *J* = 7.6, 1.5 Hz, 1H, Ar-H), 6.59 (d, *J* = 1.9 Hz, 1H, furan-H), 2.95–2.76 (m, 4H, CH_2_), 2.68 (m, 2H, CH_2_), 1.86–1.70 (m, 4H, CH_2_), 1.63 (s, 2H, CH_2_); ^13^C-NMR (CDCl_3_) δ 161.37, 157.44, 140.24, 133.49, 125.07, 123.17, 120.33, 116.19, 107.92, 53.57, 26.86, 23.71, 13.41; HRMS (ESI), *m/z* calcd for C_17_H_20_N_2_O_2_ (M+H)^+^ 285.1596, found 285.1598.

*2-Methyl-N-(2-(4-methylpiperidin-1-yl)phenyl)furan-3-carboxamide* (**5f**). Yield 84%; yellow paste; ^1^H-NMR (CDCl_3_) δ 9.06 (s, 1H, NH), 8.48 (dd, *J* = 8.0, 1.5 Hz, 1H, Ar-H), 7.33 (d, *J* = 2.0 Hz, 1H, furan-H), 7.23–7.10 (m, 2H, Ar-H), 7.04 (td, *J* = 7.6, 1.6 Hz, 1H, Ar-H), 6.56 (d, *J* = 2.0 Hz, 1H, furan-H), 2.97 (d, *J* = 11.9 Hz, 2H, CH_2_), 2.72 (dt, *J* = 6.7, 3.4 Hz, 2H, CH_2_), 1.82 (d, *J* = 14.3 Hz, 2H, CH_2_), 1.65–1.50 (m, 1H, CH), 1.35 (ddd, *J* = 15.3, 11.9, 3.9 Hz, 2H, CH_2_), 1.03 (d, *J* = 6.5 Hz, 3H, CH_3_); ^13^C-NMR (CDCl_3_) δ 161.34, 157.37, 141.88, 140.25, 133.53, 125.01, 123.12, 120.31, 118.74, 116.23, 107.86, 77.21, 76.79, 76.36, 52.91, 35.31, 30.23, 21.71, 13.42; HRMS (ESI), *m/z* calcd for C_18_H_22_N_2_O_2_ (M+H)^+^ 299.1752, found 299.1754.

*2-Methyl-N-(2-(3-methylpiperidin-1-yl)phenyl)furan-3-carboxamide* (**5h**). Yield 76%; yellow paste; ^1^H-NMR (CDCl_3_) δ 9.10 (s, 1H, NH), 8.53–8.40 (m, 1H, Ar-H), 7.32 (d, *J* = 2.0 Hz, 1H, furan-H), 7.22–7.11 (m, 2H, Ar-H), 7.06 (dd, *J* = 7.6, 1.6 Hz, 1H, Ar-H), 6.56 (d, *J* = 2.0 Hz, 1H, furan-H), 2.93 (d, *J* = 11.2 Hz, 2H, CH_2_), 2.66–2.56 (m, 1H, CH_2_), 2.43–2.28 (m, 1H, CH_2_), 1.83 (ddd, *J* = 9.8, 7.4, 2.5 Hz, 4H, CH_2_), 1.17–1.03 (m, 1H, CH), 0.94 (d, *J* = 6.5 Hz, 3H, CH_3_); ^13^C-NMR (CDCl_3_) δ 161.34, 157.44, 141.89, 140.22, 133.60, 125.07, 123.11, 120.40, 118.69, 116.21, 107.86, 77.21, 76.78, 76.36, 60.64, 53.15, 32.34, 32.29, 26.41, 19.13, 13.40; HRMS (ESI), *m/z* calcd for C_18_H_22_N_2_O_2_ (M+H)^+^ 299.1753, found 299.1754.

### 3.3. Bioassays

The six kinds of test fungi were provided by the Laboratory of Institute of Plant Protection, Chinese Academy of Agricultural Sciences (Beijing, China). After retrieval from the storage tube, the strains were incubated on potato dextrose agar (PDA) at 25 °C for several days to get new mycelia for the antifungal tests. The fungicidal activity of the target compounds was tested *in vitro* against the six plant pathogenic fungi using the mycelia growth inhibition method [22]. The tested compounds were dissolved in DMSO to prepare a 10 mg·mL^−1^ stock solution before mixing with PDA. The media containing compounds at a concentration of 50 μg·mL^−1^ were then poured into sterilized Petri dishes for initial screening. After two days at 25 °C, the colony diameter of each strain was measured. Percentage inhibition rate was calculated as (1 − a/b) × 100%, where a represents the colony diameter in the Petri dishes with tested compounds and b is the mean colony diameter in control Petri dishes. Each test was repeated three times. The 10 mg·mL^−1^ solution was diluted to 200, 100, 50, 25, 12.5, 6.25 μg·mL^−1^ and the above experiments were repeated, the inhibition rates were calculated separately. The EC_50_ values were calculated using SPSS Statistics v17.0.

### 3.4. QSAR Analyses

3D QSAR analyses were performed to predict the favorable and unfavorable moieties for improved bioactivity using the comparative molecular field analysis (CoMFA) and the comparative molecular similarity indices analysis (CoMSIA) models in the SYBYL 7.3 software [23]. CoMFA models were generated using the Sybyl 7.3 package on a Linux system. In total, 21 compounds obtained from synthesis were used to create a data set in which the bioactivity of all compounds was determined (Table 1) against *Pythium aphanidermatum* and *Rhizoctonia solani.* The pEC_50_ values were used for constructing the models. Three-dimensional molecular structures were built using the SKETCH module in Sybyl 7.3, while structural energy minimization was performed with the Tripos force field until a gradient convergence of 0.05 kcal/(mol A) was achieved. Gasteiger−Hückel charges were calculated and used to construct the CoMFA models [24].

### 3.5. Molecular Docking

Docking was performed with Surflex-Dock (SYBYL 7.3). The molecular structures were energetically minimized using Tripos with 1000 iterations and a minimum gradient of 0.005. The Gasteiger−Hückel charges of ligands were assigned. All bound water and ligands were eliminated from the protein, and the polar hydrogen atoms and the Kollman-united charges were added to the proteins.

## 4. Conclusions 

Based on bioisosterism and the computational docking experiments of commercial amide fungicides, we designed and synthesized a series of novel aromatic amides by exchanging the biphenyl group of boscalid for a nitrogen-containing heterocyclic ring. The bioassays showed that all of the target molecules exhibited considerable *in vitro* antibacterial activity against the six kinds of fungi. Compounds **1c** and **3c** with an indazolyl group exhibited higher inhibition activity than others, indicating the potential value of this moiety in agrochemical applications. A molecular docking study showed that the total docking score of compound **3c** was higher than that of boscalid and the binding of the indazolyl aniline moiety to the receptor was tighter than that of a phenylaniline in the SDH ligand binding pocket. The indazolyl group was thus very favorable for the binding of compound **3c** with SDH; although compound **3c** exhibited slightly lower activity than boscalid, indicating that improving some physical and chemical properties of compound **3c**, such as penetration, might improve its bioactivity. Further synthesis and structural optimization studies are ongoing in our laboratory.
